# Supplementary material for: Analysis of Obesity among Malaysian University Students: A Combination Study with the Application of Bayesian Structural Equation Modelling and Pearson Correlation
Source: Int J Environ Res Public Health. 2019 Feb 10;16(3):492. doi: 10.3390/ijerph16030492 (PMC6388275; doi:10.3390/ijerph16030492)
Supplement: Supplementary file 1 [file ijerph-16-00492-s001.zip › ijerph-386932-SI/ijerph-386932-Supplementary Tables.docx]

**Table 1.** Descriptive statistics for the research variables.

| **Variable** | **Category** | **Bachelor (%)** | **Master (%)** | **PhD (%)** |
| --- | --- | --- | --- | --- |
| Age | less than 21 years old | 36.7% | 24.2% | 0.0% |
|  | 21–25 years old | 45.2% | 40.2% | 0.0% |
|  | 26–30 years old | 13.1% | 25.8% | 43.9% |
|  | 31–35 years old | 4.4% | 6.3% | 29.9% |
|  | more than 35 years old | 0.6% | 3.5% | 26.2% |
| Job Experience | no job experience | 80.0% | 50.4% | 10.0% |
|  | 1–3 years | 7.3% | 24.6% | 27.7% |
|  | 4–6 years | 3.6% | 16.6% | 31.5% |
|  | 7–10 years | 0.0% | 8.4% | 9.0% |
|  | more than 10 years | 0.0% | 0.0% | 18.7% |
| Family Support | none | 0.0% | 2.9% | 17.4% |
|  | 25% of whole cost of living | 11.5% | 20.5% | 69.2% |
|  | around 50% of whole cost of living | 32.9% | 39.5% | 8.7% |
|  | around 75% of whole cost of living | 22.2% | 25.2% | 4.7% |
|  | 100% of whole cost of living | 33.4% | 11.9% | 0.0% |
| Social Media Use | less than 1 h per day | 0.0% | 0.0% | 0.9% |
|  | 1–2 h per day | 1.3% | 0.0% | 27.7% |
|  | 2–3 h per day | 16.6% | 49.6% | 58.9% |
|  | 3–4 h per day | 34.8% | 30.1% | 8.7% |
|  | more than 4 h per day | 47.3% | 20.3% | 3.7% |
| Study Time | less than 1 h per day | 0.0% | 1.2% | 5.9% |
|  | 1–2 h per day | 7.3% | 16.6% | 13.7% |
|  | 2–3 h per day | 45.6% | 40.8% | 26.5% |
|  | 3–4 h per day | 32.9% | 40.6% | 30.5% |
|  | more than 4 h per day | 14.1% | 0.8% | 23.4% |
| Sleep Duration | less than 6 h per day | 0.7% | 6.4% | 21.5% |
|  | 6–7 h per day | 21.1% | 17.4% | 62.0% |
|  | 7–8 h per day | 24.4% | 29.9% | 7.8% |
|  | 8–9 h per day | 35.7% | 36.7% | 7.2% |
|  | more than 9 h per day | 18.1% | 9.6% | 1.6% |
| Physical Activity | None | 33.2% | 18.0% | 31.8% |
|  | 1 time per week | 13.7% | 43.4% | 34.9% |
|  | 2 times per week | 23.2% | 19.9% | 8.1% |
|  | 3 times per week | 22.0% | 10.7% | 14.0% |
|  | 4 times per week | 6.6% | 6.8% | 5.0% |
|  | more than 4 times per week | 1.3% | 1.2% | 6.2% |
| Work | none | 83.9% | 78.5% | 23.7% |
|  | less than 7 h per day | 16.1% | 15.0% | 58.6% |
|  | 7–8 h per day | 0.0% | 6.4% | 8.1% |
|  | 8–9 h per day | 0.0% | 0.0% | 9.0% |
|  | more than 9 h per day | 0.0% | 0.0% | 0.6% |
| Smoking Habit | non-smoker | 72.9% | 60.2% | 47.0% |
|  | quit | 3.5% | 1.0% | 19.3% |
|  | smoker | 23.6% | 38.9% | 33.6% |
| Problems | no serious problems | 19.5% | 19.1% | 11.2% |
|  | faced 1 or 2 serious problems | 62.7% | 43.9% | 31.8% |
|  | faced more than 2 serious problems | 17.9% | 36.9% | 57.0% |
| Stress | normal | 32.6% | 58.4% | 10.3% |
|  | medium | 34.1% | 22.9% | 26.5% |
|  | high | 33.3% | 18.8% | 63.2% |
| Happiness | not happy | 23.4% | 15.0% | 34.0% |
|  | average | 44.1% | 42.8% | 33.6% |
|  | happy | 32.4% | 42.2% | 32.4% |

**Table S2.** BSEM outputs for the Bachelor, Master and PhD student groups.

| **Independent Variables** | **Beta** | **95% Credible Intervals** |
| --- | --- | --- |
| Bachelor | | |
| Source of funds → Healthy food intake | −0.39 | (−0.53, −0.11) |
| Source of funds → Mental health | 0.31 | (0.19, 0.55) |
| Source of funds → Unhealthy food intake | 0.51 | (0.38, 0.61) |
| Source of funds → Lifestyle | 0.23 | (0.06, 0.33) |
| Mental health → BMI level | 0.64 | (0.52, 0.73) |
| Mental health → Unhealthy food intake | 0.59 | (0.41, 0.66) |
| Lifestyle → Unhealthy food intake | 0.38 | (0.21, 0.46) |
| Lifestyle → BMI level | 0.67 | (0.59, 0.71) |
| Unhealthy food intake → BMI level | 0.67 | (0.49, 0.76) |
| Master | | |
| Source of funds → Healthy food intake | −0.33 | (−0.47, −0. 24) |
| Source of funds → Mental health | 0.55 | (0.37, 0.68) |
| Source of funds → Unhealthy food intake | 0.46 | (0.41, 0.55) |
| Source of funds → Lifestyle | 0.25 | (0.11, 0.39) |
| Demographics → Mental health | 0.34 | (0.22, 0.41) |
| Demographics → Lifestyle | 0.37 | (0.26, 0.43) |
| Lifestyle → Healthy food intake | 0.31 | (0.16, 0.39) |
| Lifestyle → Mental health | 0.31 | (0.22, 0.46) |
| Lifestyle → BMI level | -0.22 | (−0.31, −0.09) |
| Mental health → Unhealthy food intake | 0.33 | (0.26, 0.38) |
| Mental health → BMI level | 0.31 | (0.22, 0.46) |
| Unhealthy food intake → BMI level | 0.57 | (0.43, 0.66) |
| PhD | | |
| Source of funds → Healthy food intake | 0.42 | (0.36, 0.49) |
| Source of funds → Mental health | 0.62 | (0.58, 0.73) |
| Source of funds → Unhealthy food intake | 0.45 | (0.34, 0.55) |
| Source of funds → Lifestyle | 0.37 | (0.28, 0.42) |
| Demographics → Mental health | 0.41 | (0.35, 0.48) |
| Demographics → Healthy food intake | 0.53 | (0.44, 0.59) |
| Demographics → Lifestyle | 0.44 | (0.32, 0.52) |
| Lifestyle → Healthy food intake | 0.49 | (0.40, 0.56) |
| Lifestyle → Mental health | 0.46 | (0.33, 0.61) |
| Lifestyle → BMI level | −0.41 | (−0.55, −0.33) |
| Lifestyle → Unhealthy food intake | 0.49 | (0.38, 0.56) |
| Mental health → Unhealthy food intake | 0.36 | (0.21, 0.48) |
| Mental health → BMI level | −0.26 | (−0.38, -0.15) |
| Mental health → Healthy food intake | −0.76 | (−0.88, −0.62) |
| Healthy food intake → BMI level | −0.29 | (−0.44, −0.21) |
| Unhealthy food intake → BMI level | 0.46 | (0.32, 0.53) |
