# Supplementary material for: Analysis of Obesity among Malaysian University Students: A Combination Study with the Application of Bayesian Structural Equation Modelling and Pearson Correlation
Source: Int J Environ Res Public Health. 2019 Feb 10;16(3):492. doi: 10.3390/ijerph16030492 (PMC6388275; doi:10.3390/ijerph16030492)
Supplement: Supplementary file 1 [file ijerph-16-00492-s001.zip › ijerph-386932-SI/ijerph-386932-Text S1.docx]

**Source of Funds**

This latent variable is measured according to the following two indicators (observed variables): 1) family support and 2) income. The participants were asked how much financial support their families provided, and the possible answers were “none,” “25% of the whole cost of living,” “around 75% of the whole cost of living” and “100% of the whole cost of living.” Some students obtained funding from scholarships, working as research assistants at the university, or working outside the university. Accordingly, another question was formulated for this latent variable pertaining to the amount of monthly income (excluding family support). The possible responses were “none (no income),” “RM1000-2000 per month,” “RM2001-4000 per month,” “RM4001-6000 per month” and “more than RM6000.” [RM: Ringgit Malaysia]

**Demographics**

This latent variable is measured according to the following two main variables: (1) age and (2) work experience. The age categories were: (a) less than 21 years old, (b) 21–25 years old, (c) 26–30 years old, (d) 31–35 years old and (e) over 35 years old. The participants’ job experience was measured based on four categories: (a) no job experience, (b) 1–3 years, (b) 4–6 years, (c) 7–10 years, d) more than 10 years.

**Lifestyle**

Six indicators are the latent variables of lifestyle applied in this study, namely social media use, study time, sleep duration, physical activity, working hours and smoking habit. Average social media use (e.g., TV, mobile, tablet, etc.) per day is divided into four categories: (a) less than 1 hour per day (b) 1–2 h per day, (c) 2–3 h per day, (d) 3–4 h per day and (e) more than 4 h per day. The students were also asked to indicate the average number of hours they spent studying per day. To measure this indicator, five categories were used: (a) less than 1 hour per day, (b) 1–2 h per day, (c) 2–3 h per day, (d) 3–4 h per day and (e) more than 4 h per day. The students were asked how many hours they slept on average each day. The answers to this question were classified as: (a) less than 6 h a day, (b) 6–7 h a day, (c) 7–8 h a day, (d) 8–9 h a day and (e) more than 9 h a day. Another question asked was how many times per week (on average) the students had physical activity according to six categories: (a) none, (b) 1 time a week, (c) 2 times a week, (d) 3 times a week, (e) 4 times a week, and (f) more than 4 times a week. The fifth indicator to be measured for the students was the average number of working hours per day. The response categories were: a) none, b) less than 7 h per day, (c) 7–8 h per day, (d) 8–9 h per day and (e) more than 9 h per day. In terms of smoking habit the students’ responses were grouped as: (a) non-smoker, (b) quit and (c) smoker.

**Mental Health**

Three indicators were considered to measure mental health. These are (1) the number of serious problems faced during the last year (in this study labeled as “Problem”), (2) level of stress (“Stress”) and (3) happiness (“Happiness”). For the question about the number of serious problems the students faced in the last year, the answer categories were: (a) no serious problem, (b) 1 or 2 serious problems and (c) more than 2 serious problems. Next, the respondents described their level of stress from (a) normal, (b) medium and (c) high stress. Happiness in life was determined according to three categories: (a) not happy, (b) average and (c) happy.

**Healthy and Unhealthy Food Intake**

Unhealthy food intake entails the consumption of soft drinks, fast food, chips and sweets, whereas healthy food intake involves the consumption of whole-grain products, vegetables and fruits. Every indicator was measured using a five-point Likert-type scale. The responses obtained were ‘never,’ ‘rarely,’ ‘sometimes,’ ‘mostly’ and ‘always.’

**BMI**

BMI is calculated based on the following formula:

| $BMI \left( Metric Method \right)= \frac{(weight in kilograms)}{{height in meters}^{2}}$ |
| --- |

The Table 1 presents the BMI range for the weight categories, which are underweight, normal, overweight and obese.

**Table 1.** BMI ranges

| **BMI Range (kg/m^2^)** | **Category** |
| --- | --- |
| <18.5 | Underweight |
| 18.5–24.9 | Normal |
| 25–29.9 | Overweight |
| ≥30.0 | Obese |
